# Research advances in immune agonists and their nanoparticles for enhancing the immunotherapeutic efficacy of PD-1 inhibitors in malignancies

**DOI:** 10.3389/fonc.2025.1618903

**Published:** 2025-08-26

**Authors:** Renjie Xia, Juan Liang, Jianguo Ma, Xiaoyu Du, Liangbin Ma, Xiongxiong Han, Yong Wang, Jianwei Qin, Long Yan

**Affiliations:** ^1^ Department of Medicine, Northwest Minzu University, Lanzhou, China; ^2^ Department of Hepatobiliary Surgery, The 940th Hospital of Joint Logistic Support Force of Chinese People’s Liberation Army, Lanzhou, China; ^3^ Intensive Care Units, The 940th Hospital of Joint Logistic Support Force of Chinese People's Liberation Army, Lanzhou, China; ^4^ First School of Clinical Medicine, Gansu University of Chinese Medicine, Lanzhou, China

**Keywords:** immune agonists, PD-1 inhibitors, nanoparticle delivery systems, tumor immunotherapy, combinatorial therapy, immunotoxicity

## Abstract

Immune checkpoint blockade (ICB), particularly targeting programmed cell death-1 (PD-1), has revolutionized cancer immunotherapy but remains limited by heterogeneous therapeutic responses and immune-related toxicities. This review systematically examines the integration of immune agonists—STING, TLR, CD40, and OX40 agonists—with PD-1 inhibitors to overcome resistance and amplify antitumor immunity. Nanoparticle delivery systems emerge as transformative platforms, addressing critical limitations of free agonists, including enzymatic degradation, off-target toxicity, and poor pharmacokinetics. By leveraging tunable physicochemical properties (e.g., size, surface charge, stimuli-responsive release), nanoparticles enhance tumor-specific accumulation, prolong agonist half-life, and synergize with PD-1 inhibitors to remodel immunosuppressive microenvironments. Preclinical and early clinical studies demonstrate combinatorial strategies achieving increases in T cell infiltration and enhancements in anti-angiogenic activity compared to monotherapies. However, translational challenges persist, including nanoparticle-induced immunotoxicity (ROS-mediated inflammation), manufacturing scalability hurdles, and interspecies discrepancies in murine models. Future directions emphasize personalized nanovaccines, supramolecular cytosolic delivery systems (e.g., Calix-STING), and biomarker-driven trials to optimize efficacy in advanced pancreatic, melanoma, and immunologically quiescent tumors. This work underscores the imperative for interdisciplinary collaboration to standardize nanoparticle design and clinical validation frameworks, ultimately bridging the gap between nanomedicine innovation and oncology practice.

## 1 Introduction

Malignant tumors remain globally challenging to cure. Conventional therapies face limitations: surgery risks organ damage and misses metastases; radiotherapy incompletely eradicates tumors; chemotherapy impairs immunity and quality of life (1). With advances in understanding tumor biology, immunotherapy has gained prominence as a therapeutic strategy. Immune checkpoint blockade (ICB) has demonstrated promise in patients with malignant tumors, particularly antibodies targeting programmed cell death-1 (PD-1) and cytotoxic T-lymphocyte-associated protein-4 (CTLA-4), has demonstrated promise as a representative immunotherapy (2). Among these, PD-1 inhibitors comprise a diverse array of agents that have been extensively applied in immunotherapy for various malignancies.

However, the therapeutic efficacy of PD-1 inhibitors remains suboptimal in many malignancies. ‘Cold tumors’ exhibit limited T cell infiltration: immune-excluded types confine CD8^+^ T cells to margins, while immune-desert types lack intratumoral/peripheral CD8^+^ T cells (3). These phenotypes arise from low tumor mutational burden, diminished MHC-I expression, and reduced PD-L1 levels, compounded by immunosuppressive populations (e.g., TAMs, Tregs, MDSCs) (4). Consequently, cold tumors exhibit inherently defective antitumor immunity or ineffective T cell trafficking. As exemplified by glioblastoma (GBM)—a prototypical cold tumor—clinical trials of nivolumab or pembrolizumab failed to improve median overall survival, attributable to profound immunosuppression and spatial heterogeneity (5).

Beyond this, studies have demonstrated significant heterogeneity in treatment responses among colorectal cancer (CRC) subtypes, with PD-1 inhibitors exhibiting limited efficacy in microsatellite instability-low (MSI-L) CRC compared to improved outcomes in microsatellite instability-high (MSI-H) patients (6). PD-1 inhibitors have shown clinical benefits in only a subset of hepatocellular carcinoma (HCC) patients, while most exhibit primary resistance or rapidly develop acquired resistance (7). Furthermore, PD-1 inhibitor therapy in HCC frequently induces immune-related adverse events (irAEs), with an objective response rate (ORR) of 31% and grade 3/4 irAEs incidence of 37% observed in anti-PD-1/anti-CTLA-4 combination therapy for advanced HCC (8). Additionally, hepatotoxicity associated with PD-1 inhibitors poses a critical limitation to their long-term use. The incidence of drug-induced liver injury varies depending on agent type and dosage in HCC patients receiving PD-1 inhibitors, often manifesting as dose-dependent elevations in alanine aminotransferase (ALT) levels (9).

To enhance therapeutic efficacy while mitigating side effects and resistance without increasing PD-1 inhibitor dosage, researchers have pioneered combination therapies integrating immune agonists with ICB. Immune agonists activate the host immune system and amplify tumor immunogenicity, thereby reprogramming immunosuppressive microenvironments and promoting T cell infiltration in cold tumors— thereby potentiating PD-1 inhibitor activity.Immune agonists activate the host immune system and amplify tumor immunogenicity, thereby potentiating PD-1 inhibitor activity (10). This combinatorial strategy demonstrates potential for inhibiting HCC progression and reducing the incidence of PD-1 inhibitor resistance (11). Nevertheless, challenges such as suboptimal physicochemical stability and immune cell toxicity associated with free agonists persist. To address these limitations, nanoparticle-based delivery systems have been engineered for targeted agonist delivery, enabling precise modulation of immune cell function, reduced systemic toxicity, and specificity in immune cell activation (12). Consequently, the synergistic combination of immune agonist-loaded nanoparticles with PD-1 inhibitor immunotherapy is recognized as a novel therapeutic paradigm with significant translational potential in oncology.

## 2 Combinatorial immunotherapy: immune agonists and PD-1 inhibitors

**Figure 1:**
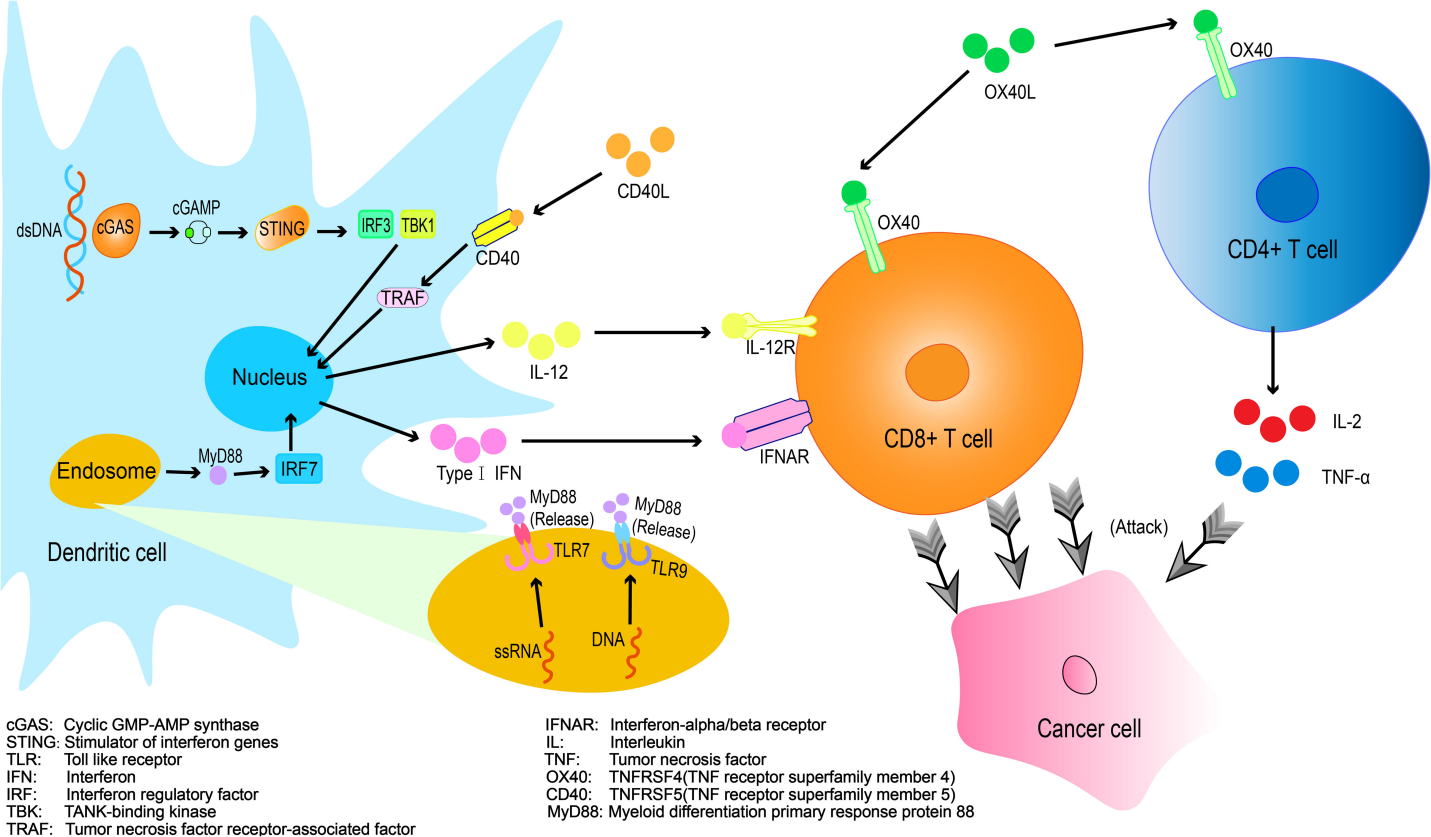
Mechanisms of immune agonists in enhancing T cell-mediated tumor killing.

Combinatorial Immunotherapy: Immune Agonists and PD-1 Inhibitors Current strategies to enhance PD-1 inhibitor efficacy include: Combination therapies with targeted agents and immune checkpoint inhibitors (ICB)、Adjunctive immune modulators、Cellular or cytokine therapies、Radiotherapy-ICB combinations (13). Among these approaches, immune agonists have demonstrated efficacy in remodeling the immunosuppressive tumor microenvironment (TME), thereby augmenting antitumor immunity and pre-conditioning the magnitude and duration of immune responses (14) (**Figure 1**).

### 2.1 Common immune agonists

#### 2.1.1 STING agonists

The stimulator of interferon genes (STING) is a multifunctional immune adaptor protein that plays a critical role in antitumor immunity (15). This pathway enhances tumor immunogenicity by upregulating MHC-I expression and tumor antigen presentation required for CD8+ T cell recognition, while concurrently promoting B cell antibody production and amplifying the functionality of dendritic cell (DC) and T cell populations (16, 17).

The combination of STING agonists with immunotherapy has emerged as a novel cancer treatment modality (18). A Phase 1 trial evaluating the STING agonist MK-1454 combined with pembrolizumab in a mixed tumor cohort demonstrated partial responses in 3 head and neck squamous cell carcinoma (HNSCC), 2 anaplastic thyroid carcinoma (ATC), and 1 triple-negative breast cancer (TNBC) patient in the combination arm—with no responses in the MK-1454 monotherapy group (0% ORR). Treatment-related adverse events (TRAEs) were reported in both arms, with no treatment-related fatalities (19). A Phase Ib study demonstrated that the STING agonist MIW815 combined with spartalizumab exhibited a favorable safety profile but modest antitumor activity in patients with advanced/metastatic solid tumors or lymphomas, achieving an ORR of 10.4%. Pharmacokinetic analysis revealed rapid absorption of MIW815 at the intratumoral injection site, with systemic exposure increasing dose-proportionally and no observed drug accumulation. Future investigations should focus on optimizing drug formulation and delivery strategies to enhance intratumoral bioavailability and therapeutic efficacy (20). Mechanistically, cGAS-STING-activated M1 macrophages recruit T cells via STING-IRF3, but concurrent STING-IRF3–signal transducer and activator of transcription 1 (STAT1) activation induces immunosuppressive PD-L1 (21). The combination of STING agonists with PD-1 inhibitors synergistically activates the cGAS-STING pathway while blocking the PD-1/PD-L1 axis, offering a promising strategy to overcome monotherapy resistance in PD-1 inhibitor-based regimens.

#### 2.1.2 TLR agonists

Toll-like receptors (TLRs), a class of pattern recognition receptor proteins, activate immune cells such as dendritic cells (DCs), T cells, and macrophages through agonist-specific recognition, playing a pivotal role in immunogenic signal transduction to promote antitumor immunity (22).

Investigators have developed combinatorial antitumor strategies integrating TLR agonists with PD-1 inhibitors. In CT26 murine models, monotherapy with the TLR7 agonist DSP-0509 achieved approximately 60% tumor growth inhibition (TGI), whereas combination therapy with anti-PD-1 antibodies enhanced TGI to >80%. Similarly, in 4T1 tumor-bearing mice, the combination regimen demonstrated significantly greater antitumor efficacy than either monotherapy. Critically, no significant reductions in body weight or other treatment-related adverse events were observed across experimental cohorts, indicating favorable tolerability profiles (23). In a Phase I/Ib trial evaluating the TLR-7 agonist LHC165 combined with spartalizumab for advanced solid tumors (including melanoma, HNSCC, and TNBC), efficacy analysis revealed an ORR of 4.8% (1/21) with monotherapy versus 8.3% (2/24) in the combination arm. Treatment discontinuation due to AEs occurred in 2 patients. Additionally, baseline biomarker analyses suggested that clinical responses to LHC165 may require a pre-existing immunologically hot TME (24). In contrast, the TLR9 agonist vidutolimod combined with PD-1 blockade demonstrated preliminary efficacy in PD-1 inhibitor-resistant melanoma patients with immunologically cold TMEs, achieving an ORR of 25% (11/44; including 7 partial responses and 4 complete responses) (25), providing critical context for patient stratification strategies. A study evaluating combined anti-PD-1 and TLR9 agonist immunotherapy in cardiac allograft-tolerant murine models demonstrated that intratumoral (i.t.) delivery achieved significantly greater suppression of tumor growth relative to systemic (i.p.) administration, without eliciting accelerated allograft rejection (26). Jiang et al. further demonstrated that the TLR9 agonist ODN1585 enhances CD8+ T cell-mediated antitumor immunity in colorectal cancer peritoneal metastasis by suppressing retinol metabolism in fibroblastic reticular cells (FRCs) and reducing Tim4+ peritoneal resident macrophage (PRM) populations, thereby potentiating anti-PD-1 therapy (27). These studies collectively demonstrate the promising therapeutic potential of TLR agonist/PD-1 inhibitor combination therapy in malignancies.

#### 2.1.3 CD40 agonists

CD40, a co-stimulatory molecule predominantly expressed on APCs such as DCs, macrophages, and B cells, transmits activation signals via interaction with CD40L on T cells to promote their activation, proliferation, and cytokine secretion. CD40 agonist antibodies mimic CD40L binding to activate CD40 signaling, enhancing APC functionality and augmenting T cell-mediated antitumor immunity (28).

A phase II trial demonstrated that the CD40 agonist sotigalimab combined with nivolumab induced durable responses in PD-1 inhibitor-resistant melanoma patients, achieving an ORR of 15.2% (5/33 partial responses) with a median duration of response ≥26 months. Critically, no treatment discontinuations due to AEs were observed among evaluable patients (29). Preclinical studies further validated this strategy: the humanized CD40 agonist KHK2840 enhanced PD-1 inhibitor efficacy in syngeneic models, significantly augmenting Th1 cells in tumor-draining lymph nodes (TDLNs), elevating DC infiltration within tumors, and concomitantly increasing intratumoral CD8^+^ T cell populations in MC-38 murine colon carcinoma models (30). In the CT2A glioblastoma murine model, combination therapy employing CD40 agonists and PD-1 inhibitors significantly enhanced survival outcomes; Mechanistically, CD4^+^ T cells crucially maintain progenitor exhausted CD8^+^ T cell populations and their responsiveness to PD-1 blockade; CD40 agonism bypasses CD4^+^ T cell dysfunction, thereby potentiating PD-1 checkpoint therapy efficacy (31). Additionally, Laurence et al. showed CD40 agonism amplifies PD-1 inhibitor efficacy in iCCA models by expanding tumor-infiltrating CD4+/CD8+ T cell populations and enhancing their activation status (32). These findings confirm CD40 agonists enhance PD-1 inhibitor responses across malignancies.

#### 2.1.4 OX40 agonists

OX40, a co-stimulatory molecule within the tumor necrosis factor receptor(TNFR) superfamily, delivers critical survival and differentiation signals that enhance T cell functionality, OX40 activation significantly amplifies the proliferation and cytokine production(including IL-2, IFN-γ, and TNF-α) in CD4+ and CD8+ T cells, augments effector functions such as granzyme B release and cytotoxic activity, promotes long-term memory cell generation, and modulates regulatory T cells (Tregs) by attenuating their immunosuppressive capacity or inducing Treg depletion (33).

In recent years, the combination of OX40 agonists with PD-1 inhibitors has emerged as a promising therapeutic strategy. Preclinical studies demonstrate that the OX40 agonist GSK3174998 synergizes with pembrolizumab to enhance antitumor immune responses in both *in vitro* and *in vivo* models, Clinically, this combination exhibits modest efficacy in immunologically ‘hot’ tumors (e.g., subsets of melanoma and NSCLC), yet demonstrates limited activity in ‘cold’ tumor microenvironments such as soft tissue sarcoma (STS) (34). This stark contrast underscores the imperative to define predictive TME biomarkers for refined patient stratification. In murine models of urothelial carcinoma, Alvim et al. reported that the triple combination of vascular-targeted photodynamic therapy (VTP), PD-1 inhibitors, and OX40 agonists achieved superior tumor growth suppression and prolonged survival compared to VTP or immunotherapy alone (35). Furthermore, Min et al. designed a phase II clinical trial (“R-ISV-RO”) integrating stereotactic radiotherapy, intratumoral injection of the RO adjuvant (a combination of TLR7 and OX40 agonists), and PD-1 blockade. Preclinical validation in urothelial carcinoma models demonstrated that triple-combination therapy (VTP + OX40 agonist + PD-1 inhibitor) significantly improved survival outcomes, achieving 60% survival at 60 days—markedly superior to monotherapy (VTP alone: 25%), dual therapies (VTP + PD-1 inhibitor: 31.25%; VTP + OX40 agonist: 20%), and untreated controls (p<0.001). This regimen significantly amplified antitumor immunity (36). Collectively, these findings provide a robust theoretical foundation for OX40/PD-1 inhibitor-based combination therapies.

### 2.2 Limitations of immune agonists

STING agonists face significant challenges in clinical translation due to their susceptibility to enzymatic degradation, short retention time, immunocyte toxicity, and inefficient cytosolic delivery (37); Additionally, they may induce epithelial barrier disruption, bronchial alveolar luminal dsDNA release, and cell death, leading to STING/type I IFN-dependent acute pulmonary inflammation (38). The STING pathway is also implicated in traumatic brain injury, spinal cord injury, subarachnoid hemorrhage, hypoxic-ischemic encephalopathy, and atherosclerosis (39, 40). Furthermore, STING agonists stimulate bone marrow-derived macrophages, triggering lipid peroxidation and cell death (41). TLR agonists are associated with adverse effects such as fever, chronic inflammation, and granuloma formation (42). In clinical trials, some TLR agonists exhibited high rates of injection site reactions (ISRs), including fever, chills, pain, and erythema (43). The pro-inflammatory cytokines and chemokines produced post-TLR activation may induce localized or systemic inflammation, contributing to irAEs (44, 45). CD40 agonists pose risks of cytokine release syndrome (CRS) and hepatotoxicity (46). Their poor tumor accumulation necessitates high doses, exacerbating toxicity while limiting efficacy (47). OX40 agonist/PD-1 inhibitor combinations demonstrate clinical responses in only a subset of patients (48). A trial evaluating the OX40 agonist MEDI0562 with immunotherapy reported frequent treatment-related adverse events (AEs) and dose-limiting toxicities (DLTs) requiring therapy discontinuation (49). Preclinical studies revealed that concurrent PD-1/OX40 agonism induces T cell apoptosis in the TME, impairing antitumor efficacy in TC-1 lung epithelial murine models, whereas delayed PD-1 inhibitor administration preserves OX40 agonist activity (50).To address these limitations, researchers have developed STING agonist-encapsulated immunostimulatory nanoparticles to enhance stability and prolong systemic retention (51, 52). Targeted delivery systems incorporating antibodies or peptides improve tumor-specific STING activation (53). Researchers have engineered nanoparticle-conjugated TLR7 agonist imiquimod to enhance therapeutic efficacy in mucosal tissues while significantly mitigating cutaneous irritation (54) (**Table 1**).

**Table 1:** Key immune agonists and their synergistic effects with pd-1 inhibitors.

Agonist Type	Mechanism of Action	Nanoparticle Delivery System	Preclinical/Clinical Outcomes
STING	Activates cGAS-STING → ↑IFN-I secretion, ↑APC function, ↑CD8+ T/NK cell infiltration	PolySTING (mesoporous silica)	- 4T1 breast tumor suppression (murine) - Avoids T cell apoptosis
TLR7/9	Binds TLR → ↑DC/macrophage activation → ↑pro-inflammatory cytokines	NS-TLR7a (silica nanoparticles)	- 4x ↑T cell infiltration (CT26 colon cancer) - 2x ↑IFN-γ production
CD40	Mimics CD40L → ↑APC-T cell interaction → ↑T cell activation	OX40L/PPT (PEG-PEI-TAT copolymer)	- ↑Survival in B16F0 melanoma (murine) - Durable responses in PD-1-resistant melanoma
OX40	Binds OX40L → ↑effector T cell proliferation, ↓Treg suppression	RO adjuvant (TLR7/OX40 co-loaded)	- Superior tumor suppression in urothelial carcinoma (triple therapy) - Delayed PD-1 dosing required

→ : Indicates progression or cascade effects in biological pathways; ↑ : Denotes enhancing/amplifying effects; ↓ : Denotes suppressing/inhibiting effects.

To address off-target toxicity and improve tumor-specific delivery, key nanoplatform design strategies include: active targeting using antibody/peptide/aptamer ligands binding tumor or TME immune cell receptors; stimuli-responsive release (pH/enzyme/ROS/reduction-triggered) for site-specific agonist activation; localized administration (intratumoral injection/hydrogels) minimizing systemic exposure; and carrier optimization via biocompatible materials, size/surface charge tuning, and sustained-release profiles to lower peak toxicity. Immunotoxicity is governed by physicochemical in nanodelivery systems may enable precise targeting, toxicity mitigation, sustained release, and synergistic immune activation, offering comprehensive solutions to overcome the current shortcomings of immune agonists.

## 3 Current research status of immunostimulatory nanoparticles

### 3.1 Characteristics of immunostimulatory nanoparticles

Diverse nanosystems provide novel drug delivery strategies to improve cancer treatment. Nanoparticle (NP)-based delivery platforms can overcome biological barriers and enhance therapeutic drug accumulation at target sites (55). Additionally, nanoscale encapsulation not only reduces systemic toxicity but also improves tumor-specific drug delivery to malignant cells (56).

In multiple tumor models (MC38 colon cancer, B16F10 melanoma, Lewis lung cancer), immunostimulatory nanoparticles (PolySTING) demonstrated superior tumor suppression compared to free cGAMP. When combined with anti-PD-1 therapy, PolySTING further enhanced antitumor efficacy. Notably, PolySTING selectively targets myeloid cells—particularly DCs—while avoiding STING-mediated apoptosis in T cells, thereby improving therapeutic safety (57). Systemically administered drugs often fail to penetrate the dense desmoplastic stroma of pancreatic tumors, leading to vascular deposition, phagocytic clearance, and inefficient tumor cell delivery (58, 59). To address this, STING/TLR4 agonist-co-loaded immunostimulatory nanoparticles were engineered to target APC-rich perivascular regions in pancreatic ductal adenocarcinoma (PDAC), effectively reversing the immunosuppressive microenvironment and inducing synergistic antitumor effects (60). Studies confirmed that intravenously delivered nanoparticles are highly internalized by perivascular macrophages at tumor sites and retained intracellularly for prolonged periods (61). The APC-enriched perivascular niche in PDAC serves as an ideal immunotherapeutic target, as nanoparticle accumulation activates and expands APCs, enhancing tumor antigen processing. Furthermore, the tunable design of immunostimulatory nanoparticles allows optimization of agonist ratios (e.g., cdGMP/MPLA), significantly boosting functional synergy and IFN-β production in murine PDAC models (60).

The enhanced permeability and retention (EPR) effect—a phenomenon where leaky tumor vasculature permits nanoparticle extravasation and retention—has long been considered the primary mechanism of nanoparticle tumor accumulation (62). However, integrated analyses of murine models, human tumors, computational simulations, and advanced imaging techniques reveal that passive diffusion through vascular gaps accounts for <3% of nanoparticle delivery. Instead, >97% of nanoparticles enter tumors via active endothelial transcytosis (63). These findings highlight the need for deeper mechanistic investigations into nanoparticle delivery pathways.

### 3.2 Common types of immunostimulatory nanoparticles for tumor therapy

#### 3.2.1 Metal ion-based nanoparticles

Manganese (Mn), an essential trace element, has been identified as an endogenous immunomodulator. Manganese ions (Mn²^+^) exhibit efficient cellular penetration, undergoing active uptake that results in significantly higher intracellular concentrations compared to extracellular levels, Under acidic conditions, MnO_2_ nanoparticles (MnO_2_ NPs) react with H_2_O_2_ to release Mn²^+^, which subsequently modulates intracellular oxidative stress and triggers diverse immune responses (64). Mechanistically, Mn²^+^ activate the cGAS-STING pathway, significantly augmenting type I interferon (IFN-I) secretion, promoting DC maturation, and enhancing the activation of CD8^+^ T cells and natural killer (NK) cells, with STING-deficient knockout models confirming pathway necessity; Furthermore, Phase I clinical trials demonstrated that Mn²^+^ combined with anti-PD-1 antibodies elicits promising therapeutic responses in patients with advanced metastatic solid tumors refractory to standard anticancer therapies (including failed combination chemotherapy, radiotherapy, or prior anti-PD-1 treatment) (n=22), achieving an objective response rate (ORR) of 45.5% (10/22) and a disease control rate (DCR) of 90.9% (20/22). These outcomes provide a robust foundation for clinical translation in malignancies including breast and ovarian cancers (65).

#### 3.2.2 Silica-based nanoparticles

Silica-based materials, owing to their high drug-loading capacity and controlled release properties, serve as ideal carriers for STING/TLR agonists. Mesoporous silica nanoparticles (MSNs), surface-functionalized with positively charged TA molecules, enable recognition and uptake by immune cells (e.g., dendritic cells and macrophages), serving as an efficient delivery vehicle for primary immune cells (66). Chen et al. engineered cdG@RMSN-PEG-TA nanoparticles that critically protect c-di-GMP from extracellular enzymatic degradation while enabling sustained intracellular release. This sequential process robustly activates the STING pathway, ultimately promoting DC, macrophage, and T cell infiltration within the TME to significantly inhibit 4T1 breast tumor growth (67). Huang et al. developed TLR7 agonist (TLR7a)-loaded silica nanoparticles (NS-TLR7a), which stimulated TLR7 signaling to amplify immune responses. In CT26 colon cancer models, NS-TLR7a increased T cell infiltration by 4-fold, doubled IFN-γ production, and demonstrated synergistic efficacy with checkpoint inhibitors, showing promise for mismatch repair-proficient (MMRp) colorectal and other MMRp cancers (68).

#### 3.2.3 Biocompatible organic nanoparticles

Low-toxicity biocompatible organic materials are widely employed for nanoparticle synthesis. Rakitina’s team engineered OX40L/PPT nanoparticles by conjugating OX40 ligand (OX40L) with a PEG-PEI-TAT copolymer (PPT). This platform enables charge-mediated complexation with plasmid DNA (pDNA), facilitating cellular uptake and permitting sustained intracellular pDNA release. The prolonged OX40 pathway activation drives persistent type I interferon production, thereby reversing tumor immunosuppression. Combined with PD-1 inhibitors, OX40L/PPT nanoparticles significantly improved survival rates in B16F0 melanoma and CT26 colon cancer models (69). Fu et al. engineered surface-modified nanocomplexes (aPD-1NCs&aOX40)@Gels that facilitate immune cell recognition and uptake. Leveraging redox-responsive properties, the platform enables rapid release of aOX40 followed by sustained release of aPD-1. This sequential release kinetics amplifies and prolongs immune activation. In B16F10 melanoma models, the nanocomplexes significantly enhanced infiltration of CD4^+^/CD8^+^ T cells, dendritic cells (DCs), and macrophages (70). Komura et al. engineered guanosine- and uridine-rich (GU-rich) hybrid hydrogels (RDgel) using DNA nanotechnology, which stabilized RNA and sustained TLR7/8 activation to amplify immunostimulatory activity (71).

#### 3.2.4 Imiquimod-loaded nanosystems

To mitigate imiquimod toxicity while enhancing efficacy, researchers have developed imiquimod-loaded nanoparticles. Dias et al. fabricated NPImq via nanoprecipitation of preformed polymers, achieving 20-fold greater anti-angiogenic activity at low doses compared to free imiquimod; furthermore, NPImq treatment significantly reduced mean papilloma diameter versus tumor-induced and placebo groups (p < 0.05) (72). Gazzi et al. formulated imiquimod-loaded polymeric nanocapsules (PEC-NCimiq) embedded in pectin hydrogels, which exhibited superior skin penetration (*particularly in the dermis with 6.8 μg deposition vs 4.3 μg for PEC-imiq*), enhanced adhesion, and controlled release to minimize side effects (73). Frank et al. engineered imiquimod nanoemulsions (NEimiq) via spontaneous emulsification, suppressing SiHa cervical cancer clonogenicity by inducing apoptosis and autophagy (74). Lapteva et al. developed imiquimod-loaded nanogels using mPEG-hexPLA polymers and carboxymethyl cellulose (CMC), demonstrating enhanced tumor targeting and therapeutic efficacy (75).

#### 3.2.5 Stimuli-responsive delivery systems

Researchers have successfully engineered pH-responsive delivery systems for immunostimulatory agonist-loaded nanoparticles that specifically target the TME. Song et al. synthesized epigallocatechin gallate (EGCG, a polyphenolic compound)- and R848 (TLR7/8 agonist)-loaded nanogels (E&R@NG) via polymerization, utilizing pH-sensitive DMAEP crosslinkers to enable acidic TME-triggered drug release. This pH-responsive immunostimulatory nanogel platform provides a synergistic immunotherapy strategy to address the limitations of immune checkpoint monotherapy (76). To overcome the poor tumor-targeting capability of CD40 agonists, Althobaiti et al. engineered low extracellular pH (pHe)-activated membrane-adhesive nanoliposomes encapsulating CD40a (pHTANL-CD40a). The system achieves pH-dependent membrane adhesion, selectively activating CD40 signaling in pancreatic tumors while minimizing systemic toxicity. *Critically, pHTANL-CD40a demonstrated significantly enhanced tumor growth inhibition (61.5% vs 27.3% for free CD40a; p<0.01)*. This approach holds significant potential to enhance the clinical efficacy of CD40 agonist/immune checkpoint inhibitor combinations in PDAC (77).

#### 3.2.6 Lipid and ester-based nanoparticles

Atukorale et al. co-encapsulated the STING agonist cyclic diguanylate monophosphate(cdGMP) and TLR4 pathway agonist monophosphoryl lipid A(MPLA) within lipid nanoparticles, which synergized with PD-1 inhibitors to enhance tumor-clearing CD8+ T cell functionality in melanoma-bearing mice (78). STING/TLR4 co-loaded lipid nanoparticles (immuno-NPs) enable systemic delivery, maintaining payload stability while reducing systemic toxicity, and demonstrate potent efficacy in murine breast cancer models with 50-60% reduction in tumor weight versus vehicle controls (79). Studies further confirmed that STING agonist-loaded lipid nanoparticles (STING-LNPs) activate hepatic macrophages to produce type I interferons (IFN-I), which mobilize systemic NK cells—particularly PD-1+ NK cells—to overcome PD-1 inhibitor resistance in B16F10 melanoma lung metastasis models (80). Local intratumoral injection of CD137/IL-2Fc-anchored liposomes reduced systemic toxicity while amplifying antitumor efficacy in B16F10 melanoma, notably achieving complete tumor regression in 60%-70% of established tumors with no observed recurrence (81). Chang et al. developed a personalized autologous nanovaccine, CpG/cGAMP-hybrid liposome-mannose (C/G-HL-Man). This vaccine enriches in lymph nodes, promotes DC-mediated antigen cross-presentation, and activates tumor-specific cytotoxic T lymphocytes (CTLs); In B16F10 murine models, C/G-HL-Man combined with fenofibrate and PD-1 antibodies suppressed recurrent melanoma progression and extended survival (82). Hanson et al. engineered PEGylated lipid nanoparticles (NP-cdGMP) encapsulating cdGMP to enhance lymphatic trafficking and drainage lymph node (dLN) accumulation. Compared to free CDNs, NP-cdGMP exhibited superior systemic dissemination, dLN retention, and CD8+ T cell activation, significantly improving antitumor outcomes (83).

#### 3.2.7 Innovative delivery strategies and engineered nanoadjuvants

Researchers designed NAcp@CD47 nanocapsules to co-deliver anti-CD47 antibodies and STING agonists into GBM tissues, where the anti-CD47 component blocks the CD47-SIRPα axis to enhance phagocytosis by macrophages and microglia, thereby reducing immunosuppression. Simultaneously, STING agonist activation promotes type-I interferon production, further amplifying antitumor immunity. This dual-action nanocapsule system reprograms the immunosuppressive microenvironment by polarizing microglia and macrophages toward M1-like tumor-associated macrophages (TAMs), ultimately reversing the ‘cold’ tumor phenotype (84). Biodegradable poly(β-amino ester) (PBAE) nanoparticles were engineered to facilitate cytosolic delivery of cyclic dinucleotides (CDNs), reducing cytotoxicity through physiological hydrolysis while enhancing nucleic acid binding efficiency, ultimately improving STING agonist-mediated cancer immunotherapy (85). Wang et al. developed c-di-AMP-loaded nanotubes (CDA-NT hydrogel) by electrostatically coupling the STING agonist with camptothecin (CPT) and the tumor-penetrating peptide iRGD. This system demonstrated potent antitumor efficacy across diverse murine tumor models, eliciting robust responses in GL-261 glioblastoma, CT26 colon carcinoma, and 4T1 breast cancer models; and induced durable immune memory. (86). Zhang et al. engineered self-assembling Ac-KLVFFAL-NH2 (KL-7) peptide nanotubes (PNT) for c-di-GMP delivery. In B16-F10 melanoma models, c-di-GMP-PNT significantly upregulated IFN-β, TNF-α, IL-6, and IL-1β expression, activated CD4+/CD8+ T cells, and enhanced tumor cell killing (87). To optimize CDN delivery, Wu’s team designed a calixarene-based supramolecular cytosolic delivery system (Calix-STING), which promoted melanoma regression, improved PD-1 inhibitor response rates, and established long-term immunological memory (88). Xian et al. chemically modified the non-nucleotide STING agonist MSA-2 with piperazine-functionalized hydrocarbon chains to synthesize nanoadjuvants. These agents robustly activated STING-mediated antitumor immunity in colorectal cancer models, reversed “cold” tumor phenotypes, and exhibited efficacy in triple-negative breast cancer, with further enhancement upon immune checkpoint inhibitor combination (89).

These studies collectively demonstrate that immunostimulatory nanoparticles amplify antitumor efficacy through enhanced antigen presentation, reversal of immunosuppressive microenvironments, and induction of memory T cell formation. By synergizing with chemotherapy, radiotherapy, and checkpoint inhibitors, these strategies establish multimodal therapeutic regimens that achieve durable antitumor immunity. Engineered agonist delivery systems represent a transformative paradigm for precision immunotherapy, particularly in advanced pancreatic cancer, melanoma, and immunologically “cold” tumors, underscoring their pivotal clinical value (**Table 2**).

**Table 2:** Classification and characteristics of immunostimulatory nanoparticles.

Nanoparticle Type	Material/Design	Key Advantages	Challenges	Applications
**Metal** **Ion-Based**	Mn²^+^-loaded NPs targeting cGAS-STING	- Endogenous immunomodulation - Activates cGAS-STING pathway, enhancing IFN-I secretion, DC maturation, and CD8^+^ T/NK cell activation - Phase I efficacy (ORR 45.5%) in advanced metastatic solid tumors	- ROS-mediated toxicity- Limited tumor penetration in dense stroma (e.g., PDAC)	Advanced metastatic tumors (breast, ovarian cancers)
**Silica-Based**	Mesoporous silica (cdG@RMSN-PE G-TA)	- High drug-loading capacity - Protects agonists from enzymatic degradation - Enables sustained intracellular release and robust STING activation - Enhances DC, macrophage, and T cell infiltration	- Manufacturing complexity - Potential silica-induced inflammation	Breast cancer (4T1 model), colon cancer
**Biocompatible Organic**	PEG-PEI-TAT copolymers (OX40L/PPT)	- Low toxicity - Charge-mediated complexation with pDNA for sustained intracellular release - Prolonged OX40 pathway activation reverses immunosuppression - Synergizes with PD-1 inhibitors	- Rapid clearance - Requires frequent dosing	Melanoma (B16F0), colorectal cancer (CT26)
**Imiquimod-Loaded**	Polymeric nanocapsules (NPImq, PEC-NCimiq), nanoemulsions (NEimiq)	- 20-fold greater anti-angiogenic activity vs. free imiquimod - Enhanced skin penetration and adhesion (dermis deposition: 6.8 µg vs. 4.3 µg) - Controlled release minimizes side effects	- Cutaneous irritation (mitigated by nanoformulations) - Toxicity at high doses	Cervical cancer (SiHa), papilloma, melanoma
**Stimuli-Responsive**	pH-sensitive nanogels (E&R@NG); pHe-activated nanoliposomes (pHTANL-CD40a)	- Tumor-specific drug release (acidic TME-triggered) - Synergy with ICB - Enhanced tumor growth inhibition (61.5% vs. 27.3% for free CD40a)	- Limited stability in systemic circulation - Complex synthesis	Immunologically "cold" tumors (e.g., PDAC)
**Lipid-Based**	STING/TLR4 co-loaded NPs (cdGMP/MPLA); CpG/cGAMP-hybrid liposomes (C/G-HL-Man)	- Systemic APC activation - Overcomes PD-1 resistance in metastasis (NK cell mobilization) - Lymph node enrichment for DC-mediated antigen cross-presentation	- High production costs - Risk of lipid peroxidation	Melanoma lung metastasis, recurrent melanoma
**Supramolecular/Engineered**	Calixarene-based cytosolic delivery (Calix-STING); Self-assembling peptide nanotubes (c-di-GMP-PNT)	- Enhanced cytosolic STING activation - Long-term immune memory - Reverses "cold" tumor phenotypes	- Scalability issues - Limited in vivo validation	Melanoma, triple-negati ve breast cancer

## 4 Potential challenges of immunostimulatory nanoparticles

### 4.1 Immunogenicity and off-target toxicity

The immune system plays a pivotal role in maintaining physiological homeostasis, and any dysregulation may disrupt this equilibrium, potentially triggering pathological states (90). Distinguishing whether immunotoxicity originates primarily from nanocarrier components or encapsulated agonists is critical for targeted risk mitigation.

Metal oxide nanoparticles induce immunotoxicity via inflammation, oxidative stress, autophagy, and apoptosis pathways (91). Immunotoxicity is governed by physicochemical properties including size, morphology, surface chemistry, and composition. Nanoparticles can activate phagocytes via receptor interactions, leading to reactive oxygen species (ROS)-mediated cell death or tumorigenesis post-uptake (92). Interactions with immune cells (e.g., macrophages, dendritic cells, neutrophils) may further exacerbate inflammatory responses and systemic toxicity (93).

### 4.2 Complex manufacturing requirements

Nanoparticle production requires high-purity materials (polymers, lipids, albumin), incurring substantial costs that escalate at scale. Furthermore, the fabrication process requires precise control over particle size, morphology, and surface properties, rendering the scale-up from laboratory to industrial production a formidable challenge that demands rigorous quality control protocols (94).

### 4.3 Barriers to clinical translation

While immunodeficient murine models are useful for evaluating immunotherapy efficacy, they fail to recapitulate the complexity of human immunity, immunocompetent models (e.g., C57BL/6 mice) better approximate human antitumor responses but exhibit interspecies discrepancies in immune regulation. Additionally, murine TMEs oversimplify human stromal complexity, limiting preclinical predictability (95).

## 5 Conclusion

Immune agonists—targeting STING, TLR, CD40, and OX40 pathways—fundamentally potentiate PD-1 blockade efficacy by reprogramming immunosuppressive tumor microenvironments, converting immunologically “cold” tumors into T cell-inflamed niches through dendritic cell activation, enhanced antigen presentation, and cytotoxic lymphocyte recruitment. Nanoparticle delivery systems critically overcome inherent limitations of free agonists such as enzymatic degradation, off-target toxicity, and poor tumor accumulation. Through targeted cytosolic release exemplified by Calix-STING supramolecular carriers, ligand-directed tumor homing via antibody conjugation, and synergistic codelivery of dual agonists in STING/TLR4 nanoliposomes, nanoplatforms establish transformative synergy with immune agonists and PD-1 inhibitors. This integrated paradigm demonstrates compelling clinical translation: TLR9 agonist vidutolimod nanoparticles achieved 25% objective response in PD-1-resistant melanoma with immunologically cold phenotypes, while Mn²^+^-STING nanoformulations combined with anti-PD-1 elicited 45.5% response in therapy-refractory metastatic tumors. Similarly, CD40 agonist sotigalimab nanoparticles induced durable remission exceeding 26 months in 15.2% of PD-1 inhibitor-resistant melanoma patients. However, this review acknowledges limitations in comprehensively covering alternative PD-1 enhancement strategies such as bispecific antibodies and oncolytic viruses, which represent significant emerging approaches in the field. Critical translational barriers persist, including nanoparticle-induced immunotoxicity from ROS-mediated inflammation, manufacturing scalability hurdles for complex nanosystems, and interspecies discrepancies in murine models that limit clinical predictability. Future efforts should prioritize toxicity-mitigated combinatorial designs, standardized biomarker validation frameworks, and personalized nanovaccines for immunoquiescent malignancies. Ultimately, the strategic convergence of immune agonist pharmacology with precision nanodelivery establishes engineered nanoparticles as pivotal tools to overcome PD-1 resistance across diverse malignancies.
